# Assessing coastal population capacity in Tsunami-prone areas: A grid-based approach

**DOI:** 10.4102/jamba.v16i1.1685

**Published:** 2024-07-30

**Authors:** Fadly Usman, Saifuddin Chalim, Fatimah Usman, Mukhamad Fathoni, Moch Rozikin, Hijrah Saputra, Keisuke Murakami

**Affiliations:** 1 Department of Urban and Regional Planning, Faculty of Engineering, Brawijaya University, Malang, Indonesia; 2 Sunan Ampel State Islamic University, Surabaya, Indonesia; 3 Faculty of Medicine, Sriwijaya University, Palembang, Indonesia; 4 Faculty of Health, Brawijaya University, Malang, Indonesia; 5 Faculty of Administrative Sciences, Brawijaya University, Malang, Indonesia; 6 Disaster Management Master’s Programme, School of Postgraduate Studies, Universitas Airlangga, Surabaya, Indonesia; 7 Faculty of Civil and Environmental Engineering, University of Miyazaki, Kibana, Japan

**Keywords:** tsunami, grid map, livelihood asset, community preparedness, hazard maps

## Abstract

Tambakrejo Beach in Blitar Regency is classified as an area that is very vulnerable to tsunami catastrophes. Many researchers have conducted studies on regions impacted by the tsunami. However, more studies into the link between the outcomes of social and spatial studies still need to be carried out because these are two different perspectives with different parameters and variables. The novel approach in this research involves delineating tsunami-affected areas and assessing population capacity in coastal regions. The hazard maps and livelihood asset variables using grid cells of a specific size have been used to identify risk levels. The grid cells used are 50 m^2^ × 50 m² so that they are expected to represent the minor units on the face of the earth, such as buildings, assets, property or land parcels, for capacity assessments or measuring the level of threat to disasters and are no longer based on regional administrative boundaries.

**Contribution:**

The research results show that using grid cells to analyse areas affected by the tsunami can provide excellent and informative results. Research findings at the research location regarding community preparedness in facing tsunamis show that communities at risk of being affected by the tsunami need to increase their capacity because the majority of communities in coastal areas, especially in the Sidorejo sub-village, have been identified as having low capacity according to several livelihood asset parameters such as financial capital in income. By increasing individual capacity, it is hoped that society will be able to avoid the threat of tsunami waves better.

## Introduction

The southern coast of the island of Java experiences a high frequency of natural disasters, such as earthquakes and tsunamis. This is because the coast is directly adjacent to the Indo-Australian fault, so a shift in the fault can trigger a tectonic earthquake and potentially cause a tsunami (Usman, Wicaksono & Setiawan 2016). Despite extensive research on the physical impacts of these disasters, there remains a significant gap in integrating social and spatial dimensions to understand and mitigate these risks comprehensively.

From January to December 2023, the number of tourists visiting Blitar Regency reached 2 833 877 people, with details of 673 foreign tourists and 2 833 201 domestic tourists. This number has increased by 9.1% compared to the year 2022. Based on the recap of data on tourist visits in 2023, Tambakrejo Beach is ranked 1st as a tourist attraction with the highest number of visitors, followed by Kampung Coklat and Serang Beach in the second and third place (Kurniawan 2024; Rizki 2024).

Tambakrejo Beach is in Blitar Regency, located in a bay about 10 km long. The jobs of most of the people around Tambakrejo Beach are fishermen, traders and farmers. To meet their daily needs, people use income from fishing. As time went by, Tambakrejo Beach began to become busy with tourists. Tambakrejo Beach, Blitar, is one of the beaches on the south coast of Java Island. Tambakrejo Beach is also a major natural tourist destination in Blitar Regency and has many tourist visits. According to the Blitar Regency tourism office, the number of tourists visiting Tambakrejo Beach, Blitar, was more than half a million in 2023, with 645 535 tourists, to be precise. Local tourists from Blitar Regency still dominate tourists who visit Tambakrejo Beach, Blitar. Still, many tourists from outside Blitar Regency visit, such as Malang, Tulungagung, Trenggalek, Ponorogo, et cetera (Zaenab 2022).

This research aims to identify areas affected by the tsunami by delineating areas potentially affected by the tsunami using elevation data in coastal areas. Using grids and cells in spatial data aims to identify the risk level of tsunami-affected areas based on elevation data and grids overlaid on top of the spatial data. This research also identifies social conditions based on livelihood asset parameters. Overlaying coastal regions prone to tsunami exposure with the results of social identification of coastal communities was then used to produce a tsunami risk map. In this research, the grid cell size used is 50 m^2^ × 50 m² with the hope that parcels and housing units can fit into the available grid cells and display more informative spatial analysis results (He et al. 2023). This approach provides a more detailed and valuable perspective. This approach comprehensively evaluates how physical risks intersect with community assets, livelihoods and preparedness measures. By combining hazard maps with livelihood asset variables in specific grid cells, a more detailed comprehension of risk levels can be achieved, facilitating the development of focused and efficient disaster response strategies.

**FIGURE 1:**
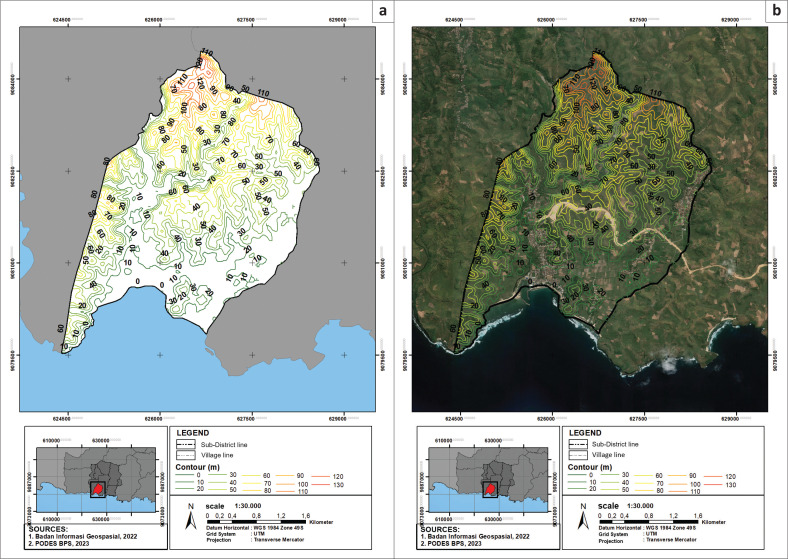
Map of Tambakrejo village, Blitar Regency, East Java Province, Indonesia. (a). Elevation map of Tambakrejo Village on the right (b). Satellite image and elevation contours of Tambakrejo Village.

Figure 1 shows the research location: Tambakrejo Village, Blitar Regency, East Java Province, Indonesia. Geographically, Tambakrejo Village is located at 8°18’52.75” South Latitude and 112°08’33.25” East Longitude, with an average air temperature of 24°C–34°C. The height in Tambakrejo Village ranges from 0 m to 125 m above sea level. Demographically, Tambakrejo Village has an area of 4.89 km², with a percentage of area to sub-district of 2.97%. The population in Tambakrejo Village is 5530 people, and the population density in Tambakrejo Village is 1130 people/km² (Faizal 2014).

The previous research shows that identifying tsunami-affected areas can be represented informatively using a grid cell system. Meanwhile, the smallest unit of social analysis, which usually uses certain administrative boundaries, such as a sub-village or village, can be presented in more detail using a grid cell system (He et al. 2023; Sun et al. 2023; Wang et al. 2023a). This research enhances our comprehensive comprehension of catastrophe risk management by integrating social and spatial studies into a unified framework. Integrating many components is essential for developing adaptable and robust communities that can survive the effects of tsunamis and other natural calamities.

## Literature review

Maps are usually used to inform land cover on the earth’s surface, such as vegetation, buildings, road networks, etc. (Farhan & Akhyar 2017; Li & Bacete 2022). Many researchers commonly use spatial data to display an area’s social, economic and cultural conditions. However, its use often displays general maps such as demographics, ethnic groups, education levels and financial capabilities. However, it refers to certain administrative boundaries, such as the level of education in villages in sub-districts or districts (Fefta et al. 2023), because the base map used always refers to efforts to simplify the results of data collection at certain administrative boundaries (Kassouk et al. 2014).

According to the UNDRR (United Nations for Disaster Risk Reduction) terminology, *disaster risk* is defined as the potential loss of life, injury or destroyed or damaged assets that can occur in a system, society or community within a certain period, which is determined probabilistically as a function of danger, exposure, vulnerability and capacity (Djalante & Garschagen 2017; Lindell 2013). Technically, this is defined through a combination of three parameters: hazard, exposure and vulnerability. When a dangerous event such as a flood, earthquake or tsunami occurs, which triggers loss of life and damage to infrastructure, this will directly impact the community and its assets, which are vulnerable to these events. When discussing disaster risk management, a disaster can highlight the following things in a community, such as the geographic area where the community lives is vulnerable to exposure to hazards or the community (including individuals) and the infrastructure, assets and property and services that may experience damage or destruction as a result. Disaster variables such as location, the strength of disaster, previous occurrences and future disaster probability are in the vulnerable group as natural hazards (Ayuningtyas et al. 2021; Fadly, Murakami & Kurniawan 2023; Ma et al. 2019; Sakamoto et al. 2020).

**FIGURE 2:**
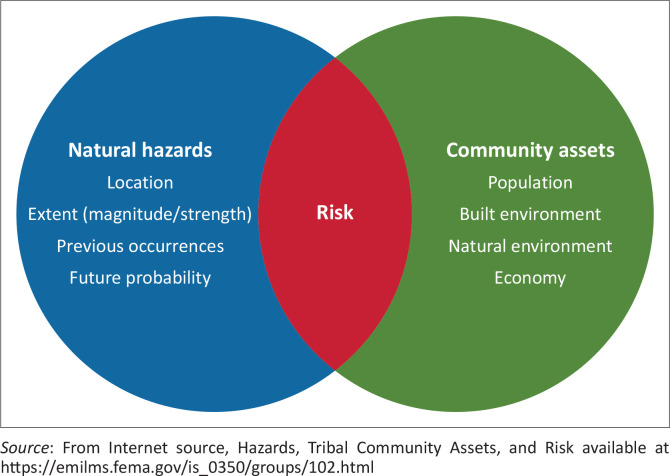
Venn diagram of the relationship between disasters, community assets and risks.

Figure 2 shows the relationship between natural hazards, community assets and risk. Risk occurs only if a disaster impacts society or assets, causing losses. If it does not cause risk, it certainly does not harm the community or loss of assets in the form of buildings, financial assets or others (Shoimah, Usman & Hariyani 2021, 2022; Usman et al. 2021). In this research, the delineation of areas prone to being affected by tsunami waves is based on elevation data. Meanwhile, to measure community assets, not only by looking at the condition of the distribution of settlements, buildings, natural environment and economic activities in Tambakrejo Village, analysis of questionnaire results with livelihood asset parameters was also carried out in this research. The administrative boundary for the research is Tambakrejo Village, Blitar, East Java Province.

However, to display the condition of the distribution of social data or the results of other social studies is to display a map based on a grid cell system with a grid size of 50 m^2^ × 50 m^2^ (He et al. 2023; Wang et al. 2023b). Several researchers integrate intelligent and green city planning approaches to contribute to urban sustainability positively. In particular, the use of participatory Geographic Information System (GIS) presents a tool to combine technological approaches in urban planning with an agenda of more equitable nature-based solutions with input from community opinions and more subjective perspectives (Maurer et al. 2023; Roy et al. 2024). For example, analysis of social vulnerability to flooding becomes an integrated part of the flood risk management process. The strategies and policies developed focus on risk-reduction methods that increase the resilience of vulnerable communities (Ajtai et al. 2023).

Academics and policymakers are increasingly aware of the importance of social capital in responding to and recovering from shocks and disasters. Using Google Maps, carrying out truth measurements and mapping social infrastructure with direct observations of location to evaluate the accuracy of available data can be used to build more real social maps (Fraser et al. 2022).

In several studies and research on livelihood assets and disasters, several researchers can show a very close correlation between each asset or capital with non-linear impacts on other capital, such as increasing social capital and developing human assets that can make a significant contribution. Significant impact on resilience to the impacts of climate change or other impacts that differ for each of the capital parameters is analysed (Daly et al. 2020; Hendratmi et al. 2022; Liu et al. 2023; Liu-Lastres et al. 2020; Nakagawa & Shaw 2004; Tufa & Megento 2022). Because the influence of each capital is so significant and closely related to community or individual preparedness efforts in facing disasters, this research also adopts a technical approach and study using parameters found in livelihood assets such as social capital, financial capital, human capital, natural capital and physical capital.

## Research methods and design

In this study, we adopted a 50 m × 50 m grid cell system to analyse disaster-affected areas and measure community assets in Tambakrejo Village, Blitar, East Java, Indonesia. Each grid cell was assigned properties and attributes based on high-resolution satellite imagery, digital elevation models (DEMs) and field surveys. These data sources helped define each cell’s physical characteristics, such as land use, elevation and proximity to critical infrastructure. To evaluate the capacity of each grid cell, we integrated physical attributes with socio-economic data. This integration involved assessing the resilience of structures based on construction type and age, economic stability from income levels and asset values and social cohesion from community surveys and demographic data. This comprehensive approach allowed us to calculate a capacity score for each grid cell, reflecting its ability to withstand and recover from tsunami impacts. The community assets include population, built environment, natural environment and economy. To define population conditions is not only based on the distribution of houses and residential areas in Tambakrejo Village but a survey is carried out using a questionnaire to determine community capacity based on several parameters such as educational background, expertise, financial ability, insurance, savings and asset ownership (livestock, houses, ponds, gardens, rice fields) (Fadly et al. 2023; Patterson, Weil & Patel 2010; Usman et al. 2023a). This research uses livelihood assets to measure community capacity based on natural, financial, human, physical and social capital.

**FIGURE 3:**
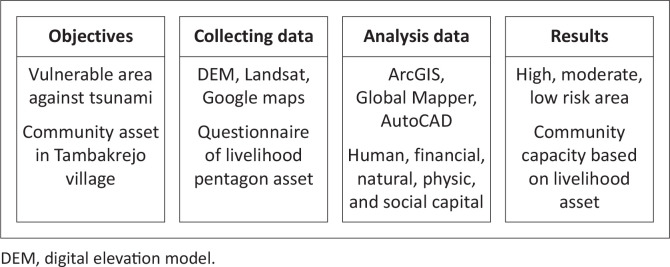
Diagram of research flow.

Figure 3 is a research flow diagram comprising two study groups, namely studies of areas prone to being affected by the tsunami disaster using a spatial approach and community asset analysis using livelihood asset parameters to produce a social map based on the livelihood asset pentagon. Analysis of community assets using variables in the livelihood pentagon assets, including human capital (education, skills, knowledge, abilities and capabilities), natural capital (forest, river, ponds, trees, land), financial capital (income, credit, saving, investment), social capital (fisherman community, culture and religion) and physical capital (wave barrier construction, shelter, access road, infrastructure, health care facilities) (Fischer et al. 2021; Islam & Walkerden 2022; Usman et al. 2016, 2023b; Vecere et al. 2017).

The close relationship between the use of geospatial maps and disaster mitigation efforts has been widely carried out by disaster researchers. The results show that the use of data and spatial analysis is beneficial in making policies or technical efforts to determine integrated, fast, comprehensive and efficient disaster-management strategies (Ekaputra et al. 2022; Manfré et al. 2012; Mizutori 2020). Spatial data can clearly show areas affected by disasters or areas that are safe from disasters; so spatial data analysis in reducing disaster risk should be carried out before a disaster occurs as a mitigation measure (Appleby-Arnold et al. 2018; Gibson et al. 2016; Han et al. 2019; Ikeda & Inoue 2016; Kosaka et al. 2023; Norris et al. 2002; Shimura & Yamamoto 2014).

**FIGURE 4:**
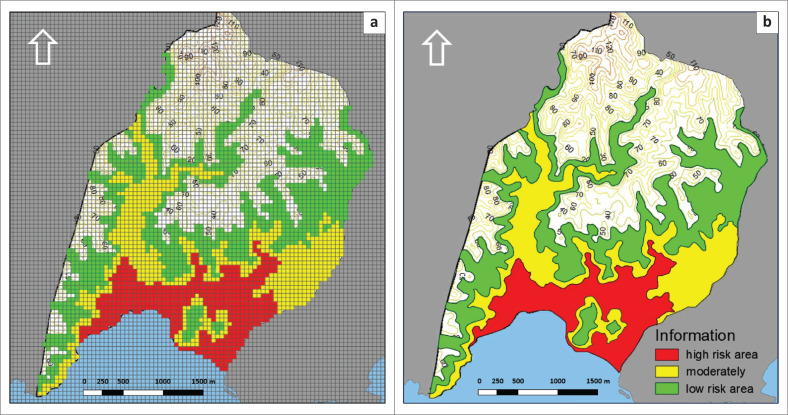
Difference between (a) grid cell system and (b) GIS delineation with digital elevation model analysis.

Figure 4 shows the differences in using a grid cell system and delineating disaster-prone areas using spatial analysis at the research location, namely in Tambakrejo Village, Blitar Regency, East Java. Both use primary data from the DEM, which then creates elevation intervals that represent three risk groups, namely the high-risk group with an elevation of 0 m – 10 m above sea level, the medium-risk group with an elevation of 10 m – 30 m above sea level, while those included in the low risk category with an elevation of 30 m – 50 m above sea level (Fadly & Murakami 2012; Joyce et al. 2009; Mahiddin, Affandi & Mohamad 2021; Valachamy et al. 2022).

Using spatial data and three-dimensional models in spatial analysis can display precise locations that can be used as shelters or temporary shelters when a disaster occurs (Xu et al. 2023; Zou et al. 2023). However, in this research, spatial analysis is limited to knowing the areas affected by the tsunami disaster in low-risk, moderate and high-risk areas. Meanwhile, the determination of the distribution of shelters is only based on community opinion, which was studied based on a questionnaire filled out by 578 respondents, because the local government has determined the distribution of shelters as evacuation places in the event of a tsunami wave in Tambakrejo Village, Blitar.

### Ethical considerations

An application for full ethical approval was made to the Brawijaya University’s Research Ethics Committee and ethics consent was received in 2023. The ethics approval number is 43/UNl0/PN/2023.

## Results

**FIGURE 5:**
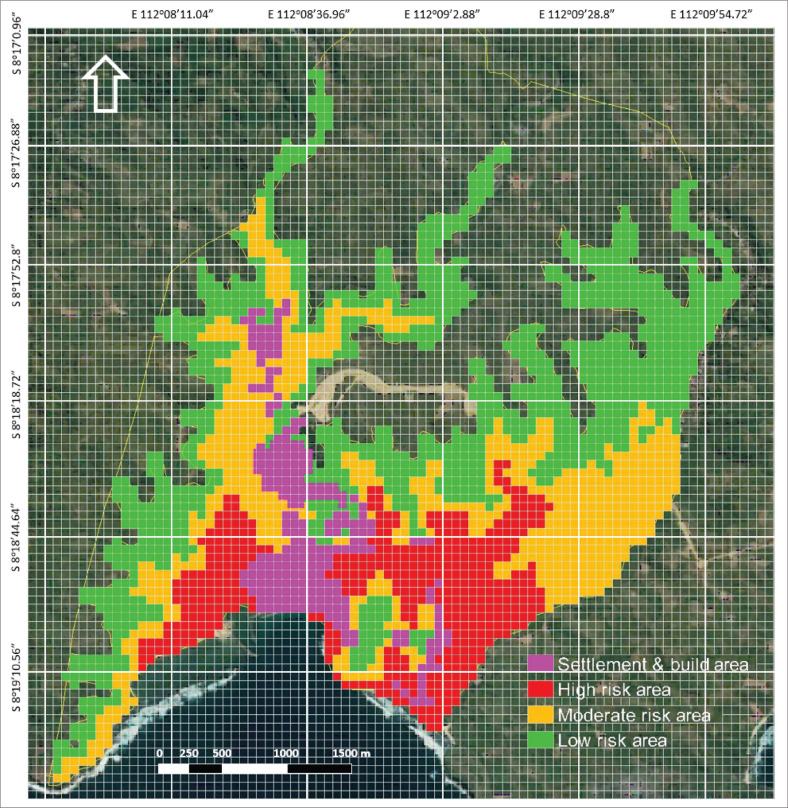
Affected settlements and built-up areas based on the grid cell system.

If Figure 4 shows the differences in analysis results using DEM spatial data converted into grid system form, Figure 5 is the result of overlaying three risk groups in grid system format in an area with land cover in the form of a built-area in Tambakrejo Village, Blitar Regency. Most of the built-up areas are high-risk areas, which directly impacts the strategic steps that must be taken to prevent significant losses if a tsunami disaster occurs in Tambakrejo Village. When looking at the size of a 50 m^2^ × 50 m² grid on a map, this grid size is still perceived as too large to identify the affected building units.

Breaking down the built-up areas into smaller grid units makes pinpointing specific structures at risk during a tsunami event easier. This level of detail allows for more targeted evacuation plans and disaster response strategies to be implemented in Tambakrejo Village. Additionally, by incorporating DEM spatial data into the grid system analysis, the area’s topographical features can be considered, further enhancing the accuracy of risk assessments.

When exploring the impact of built-up areas being classified as high risk, one key aspect is the effectiveness of current disaster prevention and response strategies. With the identification of specific structures at risk in Tambakrejo Village, authorities can tailor evacuation plans to ensure the safety of residents in those areas. Additionally, by incorporating topographical features into the analysis, emergency response teams can better understand the potential obstacles and challenges they may face during a tsunami event. This level of detailed planning and preparation is crucial in minimising the impact of disasters and thus saving lives.

Table 1 depicts the number of grids in each category of affected area, which refers to the map in Figure 5. With a 50 m^2^ × 50 m² grid, it is difficult to differentiate land cover in buildings into certain categories, such as private buildings, public buildings or other property assets. So, an explanation of the use of grid cells can be seen in Table 1 shows the number of buildings at risk of being affected by tsunami waves based on three risk groups: high-risk area, moderate and low-risk area.

**TABLE 1:** Number of grid cells in a vulnerable area.

No	Tsunami affected categories	Vulnerable area		Build area	
		Grid	Area (m^2^)	Grid	Area (m^2^)
1	High-risk area	553	13.825	105	2.625
2	Moderate risk area	815	20.375	87	2.175
3	Low-risk area	1.150	28.750	55	1.375

m^2^, square meters.

Table 1 shows the number of grids and area size for each risk level in Tambakrejo Village’s vulnerable areas. Using a 50 m^2^ × 50 m² grid has simplified the calculation of the area affected because the calculation is based on the number of grids on the map. Table 1 refers to Figure 5, a map of the distribution of buildings and settlements at risk of being affected by the tsunami disaster. Based on Table 1, the area with a high-risk level is quite large, namely 13 825 m^2^ or 1.38 Ha, and the area with a medium risk level is 20 375 m² or 2.03 Ha, while the area with a low-risk level is 28 750 m² or 2.87 Ha. Meanwhile, the built-up area with a high-risk impact is 105 grids or around 2625 m^2^, and the built-up area with medium risk is 87 grids or an area of 2175 m², while the low-risk area is 55 grids or an area of 1375 m².

Using maps in grid form aims to obtain values in grid cells directly, namely by representing appropriate spatial data on a local and regional scale, then continuing by using the cross-check method and conventional measurements on land (Sun et al. 2023). The risk assessment method using spatial data can select and reflect targets or predictions of damage that arise from being directly affected by disasters. The indexation method using a grid map can increase the accuracy of raw data distribution, thereby minimising distortion of the disaster risk index, which is calculated manually using a computer. Disaster risk assessment on spatial data is based on an indicator-based approach, namely by using disaster risk maps and thematic data that use specific parameters for further analysis (Wang et al. 2023a). In this research, the grid cells on the map are given values based on elevation data and real conditions in the field to produce a map of areas at risk of being affected by disasters.

Table 2 shows the results of data collection based on a questionnaire filled out by 578 respondents who are residents of Tambakrejo Village. Table 2 is the result of community asset analysis using variables in the livelihood pentagon asset. Tambakrejo Village, Blitar, consists of two sub-village administrative areas: Krajan and Sidorejo. In this research, the pentagon asset graph is also differentiated based on the distribution of respondents in the two sub-villages. The results of the respondent data recapitulation show that the Krajan sub-village residents have better capacity even for each variable and sub-variable. For example, the financial capital in Krajan shows that residents in Krajan are more prosperous than Sidorejo in terms of income per capita, credit, savings, investment and insurance. Meanwhile, regarding the values of social capital variables such as cultural and religious parameters, both residents in Krajan and Sidorejo have social capital values that are not much different, except for the fisherman community, which is dominated by residents who live in coastal areas such as in the Krajan sub-village. The value that is quite significantly different between Krajan and Sidorejo is human capital. The average education, skill, ability, capability, training and knowledge in the Krajan sub-village is better than that of residents in the Sidorejo sub-village.

**TABLE 2:** Livelihood asset in Tambakrejo village, Blitar, East Jawa Province.

Capital	Sub variables	Score	
		Krajan	Sidorejo
Natural	Forest area	4.91	2.87
	River	2.50	3.95
	Ponds	4.87	1.05
	Trees	4.12	4.57
	Land area	2.55	3.91
Financial	Income per capita	4.55	2.35
	credit	3.32	2.20
	Insurance	2.20	1.95
	Saving	4.81	3.71
	Investment	4.82	3.58
Human	Education	3.82	3.31
	Skill	3.80	3.52
	Ability and capability	4.91	3.15
	Training and knowledge	4.50	2.75
Physic	Wave barrier construction	3.20	2.32
	Access road	4.52	4.25
	Health facility	4.25	3.88
	Shelter	4.87	4.38
	Infrastructure	4.85	3.51
Social	Fisherman community	4.82	2.35
	Culture	4.82	4.52
	Religion	4.91	4.33

**FIGURE 6:**
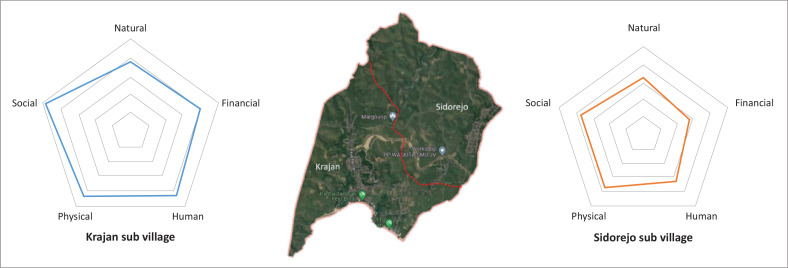
Form of livelihood pentagon asset for Krajan and Sidorejo sub-village.

Figure 6 shows the administrative boundaries of the Krajan sub-village and Sidorejo sub-village with the results of calculations on the livelihood pentagon assets for each sub-village. Figure 6 refers to Table 2 and Table 3, representing the population capacity in each sub-village based on the total variable and sub-variable values in livelihood assets in Tambakrejo Village. The total capacity value in the Krajan sub-village is 21.18, while in the Sidorejo sub-village, it is 16.61. This significant difference occurs in all sub-variables, such as natural, financial, human, physical and social capital.

**TABLE 3:** The capacity of sub-villages in Tambakrejo.

No	Sub villages	Natural	Financial	Human	Physical	Social	Capacity
1	Krajan	3.79	3.94	4.26	4.34	4.85	**21.18**
2	Sidorejo	3.27	2.76	3.18	3.67	3.73	**16.61**

Referring to Table 3, strategies for increasing population capacity in the Sidorejo sub-village can be mapped by focusing on the values that are still lacking, such as making efforts to increase human resources so that it will have a direct impact on increasing other capital, such as financial and social capital. Increasing population capacity is urgently needed so that people or individuals living in Tambakrejo Village, Blitar, can always increase their preparedness for dealing with the threat of tsunami waves.

**FIGURE 7:**
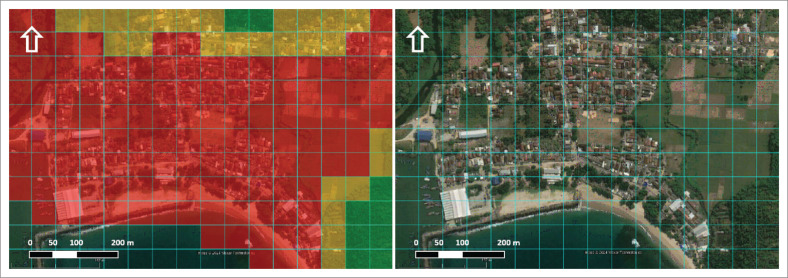
Settlement and build areas affected are based on a grid cell system.

Spatial analysis using a 50 m^2^ × 50 m² grid resulted in less than satisfactory results. Figure 7 shows how a 50 m^2^ × 50 m² grid looks too large to identify land parcels or the distribution of houses in the research location.

**FIGURE 8:**
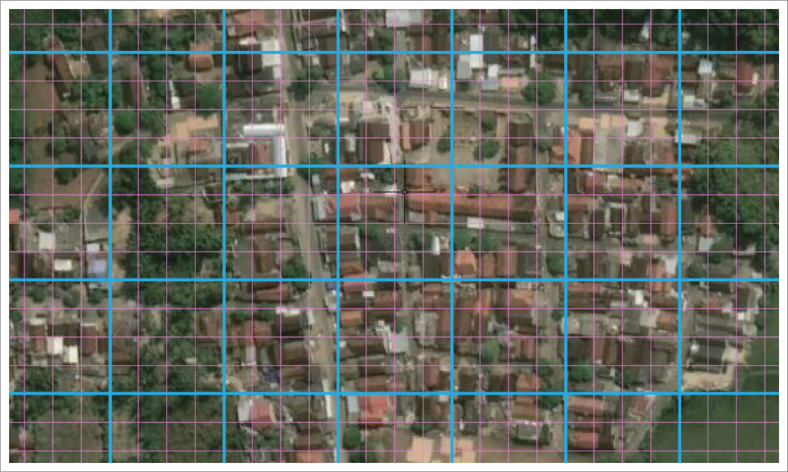
The difference in size is 50 × 50 and 12.5 × 12.5 in the grid cell system.

In Figure 8, the blue grid shows a 50 m^2^ × 50 m² grid size, while the cyan grid measures 12.5 m^2^ × 12.5 m². In fact, the smaller the grid size can represent the smallest units on the map, such as land cover, houses, buildings, financial assets or others, so that each smallest unit can have a different colour, such as red for high-risk, yellow for medium risk and green for low-risk assets.

Figure 8 shows the difference between grid cells measuring 12.5 m^2^ × 12.5 m² (cyan line) and 50 m^2^ × 50 m² (blue line). This study found that using a smaller grid measuring 10 m^2^ × 10 m² is better than 50 m^2^ × 50 m². The small grid cells have been shown to represent land cover more accurately and in a more informative way. However, it should be noted that a smaller grid size certainly means that each grid cell’s data analysis and assessment process will require longer research time but with more detailed results.

## Discussion

Using a 50 m^2^ × 50 m² grid system in spatial analysis has underscored the significant delineation of high-risk tsunami areas in Tambakrejo Village, revealing a pronounced vulnerability in built-up regions. The comparative analysis of community assets between Krajan and Sidorejo sub-villages illustrated marked disparities, highlighting Sidorejo’s lower capacity across multiple asset categories, directly influencing its resilience to tsunamis.

The discerned high-risk areas within Tambakrejo’s built environment resonate with broader literature emphasising the need for precise risk localisation in tsunami-prone zones. This study’s findings contribute to the nuanced understanding of risk variation within small geographical confines, aligning with and expanding upon previous research that advocates for granular risk assessment methodologies. Furthermore, the disparities in community asset strengths between sub-villages align with studies emphasising socio-economic factors in disaster resilience, underscoring the critical need for community-specific disaster preparedness strategies.

A significant strength of this study is the innovative application of a grid-based spatial analysis that enhances the precision of risk mapping in coastal settings. However, the chosen grid size (50 m^2^ × 50 m²) limits the ability to detail individual structures’ vulnerability, suggesting that finer resolutions could provide more actionable insights. While this study provides a robust starting point for localised risk assessment, its findings are constrained by the spatial resolution’s adequacy and the inherent complexities in extrapolating grid-based analyses to real-world disaster response scenarios.

The findings underline the imperative for targeted disaster risk reduction strategies that account for intra-community variations in vulnerability and capacity, especially in heterogeneous environments like Tambakrejo Village. For policymakers and practitioners, this study advocates for adopting more granular spatial analyses in disaster preparedness planning, particularly in areas with significant socio-economic diversity. Future research should explore the application of finer grid sizes in risk assessment and incorporate comprehensive socio-economic data to enhance predictive accuracy and practical utility. Additionally, the study underscores the importance of integrating community-specific asset evaluations into disaster risk frameworks, ensuring that mitigation strategies are both nuanced and contextually relevant.

## Conclusion

Analysis of areas at risk of being affected by a tsunami using grid cells can display more informative spatial data because each grid represents a specific size, making it easier to calculate the size of the affected area based on the number of grids identified. Indexation on the grid can also be simplified. In this study, indexation focuses on the level of tsunami disaster risk, which is categorised into three categories, namely high risk in red with an area of 13 825 m², medium risk in yellow with an area of 20 375 m² and low risk in green with an area 28 750 m².

The grid cell system is also used to identify land cover in the form of built-up areas that are at risk of being affected by a tsunami based on three tsunami disaster risk categories, with the built-up area at high risk being 2625 m², the area at medium risk being 2175 m² and the built-up area at low risk being 1375 m². An interesting finding from this research is that the grid cell size of 50 m^2^ × 50 m² is too large to be able to identify more specific land cover, such as the type of building, type of land cover in the form of vegetation or the quality of the building and the socio-cultural conditions of the building owner. So, the questionnaire results from 578 respondents from residents of Tambakrejo Village can only be displayed in the form of a pentagon asset diagram. Tambakrejo Village consists of two sub-villages where the overall capacity value for the Krajan sub-village is 21.18, and Sidorejo sub-village is 16.61, where the total value in Sidorejo is corrected quite significantly on the two sub-variables of livelihood assets, namely human capital and financial capital.

Further research will be more informative if we use a grid cell smaller than 50 m^2^ × 50 m² because the size of this grid cell is too large to represent land cover more specifically and informatively. The grid cell size of 10 m^2^ × 10 m² can be used as an acceptable size to display more specific and informative land cover and can even represent the social conditions in each grid in more detail.
