# Erratum: Assessing coastal population capacity in Tsunami-prone areas: A grid-based approach

**DOI:** 10.4102/jamba.v16i1.1770

**Published:** 2024-09-05

**Authors:** Fadly Usman, Saifuddin Chalim, Fatimah Usman, Mukhamad Fathoni, Moch Rozikin, Hijrah Saputra, Keisuke Murakami

**Affiliations:** 1 Department of Urban and Regional Planning, Faculty of Engineering, Brawijaya University, Malang, Indonesia; 2 Sunan Ampel State Islamic University, Surabaya, Indonesia; 3 Faculty of Medicine, Sriwijaya University, Palembang, Indonesia; 4 Faculty of Health, Brawijaya University, Malang, Indonesia; 5 Faculty of Administrative Sciences, Brawijaya University, Malang, Indonesia; 6 Magister Disaster Management, Postgraduate School, Universitas Airlangga, Surabaya, Indonesia; 7 Faculty of Civil and Environmental Engineering, University of Miyazaki, Kibana, Japan

In the published article, Usman, F., Chalim, S., Usman, F., Fathoni, M., Rozikin, M., Saputra, H., Murakami, K. et al., 2024, ‘Assessing coastal population capacity in Tsunami-prone areas: A grid-based approach’, *Jàmbá: Journal of Disaster Risk Studies* 16(1), a1685. https://doi.org/10.4102/jamba.v16i1.1685, there was an error in affiliation 6.

Instead of:

‘Disaster Management Master’s Programme, School of Postgraduate Studies, Universitas Airlangga, Surabaya, Indonesia’,

It should be:

‘Magister Disaster Management, Postgraduate School, Universitas Airlangga, Surabaya, Indonesia’.

The publisher apologises for this error. The correction does not change the study’s findings of significance or overall interpretation of the study’s results or the scientific conclusions of the article in any way.

